# Tumor Necrosis Factor-Alpha and the ERK Pathway Drive Chemerin Expression in Response to Hypoxia in Cultured Human Coronary Artery Endothelial Cells

**DOI:** 10.1371/journal.pone.0165613

**Published:** 2016-10-28

**Authors:** Su-Kiat Chua, Kou-Gi Shyu, Yuh-Feng Lin, Huey-Ming Lo, Bao-Wei Wang, Hang Chang, Li-Ming Lien

**Affiliations:** 1 Graduate Institute of Clinical Medicine, College of Medicine, Taipei Medical University, Taipei, Taiwan; 2 Division of Cardiology, Department of Internal Medicine, Shin Kong Wu Ho-Su Memorial Hospital, Taipei, Taiwan; 3 Division of Nephrology, Department of Medicine, Shuang Ho Hospital, Taipei Medical University, New Taipei, Taiwan; 4 Central Laboratory, Shin Kong Wu Ho-Su Memorial Hospital, Taipei, Taiwan; 5 Department of Emergency Medicine, Shin Kong Wu Ho-Su Memorial Hospital, Taipei, Taiwan; 6 Institute of Injury Prevention and Control, College of Public Health, Taipei Medical University, Taipei, Taiwan; 7 School of Medicine, College of Medicine, Taipei Medical University, Taipei, Taiwan; 8 Department of Neurology, Shin Kong Wu Ho-Su Memorial Hospital, Taipei, Taiwan; Centro Cardiologico Monzino, ITALY

## Abstract

**Background:**

Chemerin, a novel adipokine, plays a role in the inflammation status of vascular endothelial cells. Hypoxia causes endothelial-cell proliferation, migration, and angiogenesis. This study was aimed at evaluating the protein and mRNA expression of chemerin after exposure of human coronary artery endothelial cells (HCAECs) to hypoxia.

**Methods and Results:**

Cultured HCAECs underwent hypoxia for different time points. Chemerin protein levels increased after 4 h of hypoxia at 2.5% O_2_, with a peak of expression of tumor necrosis factor-alpha (TNF-alpha) at 1 h. Both hypoxia and exogenously added TNF-alpha during normoxia stimulated chemerin expression, whereas an ERK inhibitor (PD98059), ERK small interfering RNA (siRNA), or an anti-TNF-alpha antibody attenuated the chemerin upregulation induced by hypoxia. A gel shift assay indicated that hypoxia induced an increase in DNA-protein binding between the chemerin promoter and transcription factor SP1. A luciferase assay confirmed an increase in transcriptional activity of SP1 on the chemerin promoter during hypoxia. Hypoxia significantly increased the tube formation and migration of HCAECs, whereas PD98059, the anti-TNF-alpha antibody, and chemerin siRNA each attenuated these effects.

**Conclusion:**

Hypoxia activates chemerin expression in cultured HCAECs. Hypoxia-induced chemerin expression is mediated by TNF-alpha and at least in part by the ERK pathway. Chemerin increases early processes of angiogenesis by HCAECs after hypoxic treatment.

## Introduction

Adipose tissue acts as an endocrine organ that produces and secretes multiple immunomodulatory proteins known as adipokines [1]. The number of known members of the adipokine family continues to grow, including leptin, adiponectin, apelin, interleukin 6 (IL-6), and chemerin [2]. Some of these adipokines are pleiotropic cytokines, which participate in inflammation and immune regulation [3]. Studies have shown that these adipokines are involved in the pathogenesis of many cardiovascular diseases including ischemic heart disease, cardiomyopathy, and heart failure [3, 4]. Different adipokines may indicate various prognoses in heart failure [5, 6].

Chemerin, also known as tazarotene-induced gene 2 protein (TIG2) or retinoid acid receptor responder 2 (RARRES2), is secreted mainly in the liver and adipose tissue as an inactive 18-kDa propeptide (prochemerin) and is subsequently cleaved by a protease in the C-terminal domain, with formation of a 16-kDa active protein [7]. Chemerin is now known to participate in the metabolism, regulation of immune responses, tissue inflammation, injury, and differentiation into adipose cells [8]. One study showed that chemerin is associated with adipogenesis regulation and metabolic syndrome; the latter means the elevated body–mass index, and insulin resistance [9]. In addition, chemerin was found to be associated with impaired left ventricular systolic function, suggesting that chemerin is closely associated with ischemic heart disease, acute coronary syndrome, and heart failure [10, 11]. Chemokine like receptor 1 (CMKLR1), a G_i_ protein-coupled chemerin receptor is highly expressed in vascular endothelial cells and is regulated by several proinflammatory cytokines, including tumor necrosis factor-alpha (TNF-alpha) and IL-6 [12]. Taken together, these data suggest that chemerin may be an independent adipocytokine marker of the inflammatory state of vascular endothelial cells and cardiovascular disease [13].

Various studies have shown that hypoxia can cause cardiomyocyte degeneration, apoptosis, and fibrosis, which may lead to cardiomyocyte hypertrophy, myocardium remodeling, and heart failure [14, 15]. Endothelial cells in the coronary arteries form the inner surface of the vessels and drive a response to hypoxic conditions in ischemic heart disease, including coronary atherosclerotic disease or acute coronary syndrome [16]. In response to hypoxic conditions, a number of genes are upregulated by signaling cascades, such as p38 mitogen-activated protein kinase (MAPK), Jun N-terminal kinase (JNK), hypoxia induce factor-1alpha (HIF-1alpha) and ERK, in vascular endothelial cells [17–20]. These responses include vascular-endothelial-cell proliferation, migration, and subsequently angiogenesis, but also growth arrest and apoptosis. Thus, it is important to understand the molecular mechanisms triggered by exposure of vascular endothelial cells to hypoxia.

The effects of hypoxia on chemerin expression and its molecular regulation in the course of angiogenesis in human coronary artery endothelial cells (HCAECs) have not been studied yet. The detailed mechanisms of action of chemerin during hypoxia may provide new insights into the therapeutic strategies against ischemic heart disease or acute coronary syndrome. Therefore, we hypothesized that hypoxia can activate a proinflammatory process that may regulate the expression of chemerin in HCAECs. In this regard, the aim of this study was to elucidate 1) the expression of chemerin in hypoxic HCAECs, 2) the possible molecular mechanism underlying the changes in chemerin expression and its molecular regulation in hypoxic HCAECs.

## Methods

### Primary Culture of HCAECs and Hypoxia Settings in the Incubator

HCAECs, obtained from PromoCell GmbH (Heidelberg, Germany), were cultured in the endothelial cell growth medium containing 10% of fetal bovine serum, 100 U/mL penicillin, and 100 μg/mL streptomycin. The cells were grown in a humidified atmosphere containing 5% of CO_2_ at 37°C.

For hypoxic stimulation, a humidified temperature-controlled incubator (ProOX model 110; BioSpherix, Ltd., Redfield, NY, USA) was used. HCAECs were cultured under various hypoxic conditions: (i) 2.5% O_2_, 5% CO_2_, and 92.5% N_2_; (ii) 5% O_2_, 5% CO_2_, and 90% N_2_; and (iii) 10% O_2_, 5% CO_2_, and 85% N_2_ for 1, 2, 4, or 6 h or longer. A lower oxygen concentration (1%) was attempted; however, the cultured HCAECs had difficulty surviving such severe hypoxia. As a control, HCAECs were cultured at 5% CO_2_ in air.

### Western Blot Analysis

This analysis was performed as previously described [21]. Monoclonal rat anti-human chemerin and anti-ERK antibodies (Santa Cruz Biotechnology, Paso Robles, CA) were used. Equal protein loading of the samples was verified by incubating with a monoclonal anti-α-tubulin antibody (Sigma, St Louis, MO). Proteins were incubated with specific antibodies (1:200 dilution) for 1 h at room temperature followed by incubation with a 1:10000 dilution of horseradish peroxidase-conjugated polyclonal anti-goat IgG antibody for 1 h at room temperature. Signals were visualized by chemiluminescence detection. Densitometry was used for quantification of all western blot data.

### Quantitative Real-Time Reverse Transcription Polymerase Chain Reaction

Total RNA was isolated from HCAECs using the single-step acid guanidinium thiocyanate/phenol/chloroform extraction method according to the protocol provided by the manufacturer. Real-time PCR was performed as previously described [21]. The chemerin primers were 5’-GGAAGAAACCCGAGTGCAAA-3’ and 5’-ACCAACCGGCCCAGAACT-3’.

### RNA Interference

HCAECs were transfected with 800 ng of ERK, short interfering RNA (siRNA), chemerin siRNA or HIF-1alpha, which target a specific 19–21 nt sequence to knock down gene expression (Dharmacon Inc., Lafayette, CO). The ERK sense and antisense siRNA sequences were 5’-GACCGGAUGUUAACCUUUA-3’ and 5’-UAAAGGUUAACAUCCGGUC-3’, respectively. Chemerin sense and antisense siRNA sequences were 5’-GCAUCAAACUGGGCUCUGA-3’ and 5’-UCAGAGCCCAGUUUGAUGC-3’, respectively. HIF-1alpha sense of siRNA sequence and antisense of siRNA sequence 5'-UGAGAGAAAUGCUUACACA-3' and 5'-UGUGUAAGCAUUUCUCUCA-3', respectively. (Santa Cruz Biotechnology)

As a negative control, scramble siRNA and green fluorescent protein siRNAs were used (Dharmacon Inc., Lafayette, CO). HCAECs were transfected with the siRNA oligonucleotides using the Effectene Transfection Reagent Kit (Qiagen Inc., Valencia, CA, USA). After incubation at 37°C for 24 h, HCAECs were subjected to hypoxia and then western blot analysis.

### Electrophoretic Mobility Shift Assay (EMSA)

The EMSA was performed as previously described [22]. Briefly, a Bio-Rad protein assay kit was used to determined nuclear protein concentrations in HCAECs. Consensus and control oligonucleotides (Santa Cruz Biotechnology) were labeled by polynucleotide kinase to incorporate [γ-^32^P]ATP. The consensus oligonucleotide sequence for transcription factor SP1 was consensus 5’-ATTCGATCGGGGCGGGGCGAGC-3’. The SP1 mutant oligonucleotide sequence was 5’- ATTCGATCGGTTCGGGGCGAGC-3’. Controls were set up in each group with mutant oligonucleotides or cold oligonucleotides to compete with the radiolabeled oligos.

### Chemerin Promoter Activity Assay

The -1400 to -900 region of the rat chemerin promoter was used to generate a plasmid construct. Briefly, chemerin genomic DNA was amplified with the forward primer, 5’-AGCCTCGGAGAAAGCCTGGCT-3’ and reverse primer, 5’-TTGAGGATTAAATGAAGAAACATGAA-3’. The amplified genomic product was digested with M1uI and Bg1II restriction enzymes. The DNA fragment was then ligated into the pGL3-basic luciferase plasmid vector (Promega) digested with the same enzymes. The chemerin promoter contains a conserved SP1-binding site between positions ‒1137 and ‒1136. For the mutant construct, the SP1-binding site was mutated using a mutagenesis kit (Mission Biotech, Taipei, Taiwan). The mutations were confirmed by DNA sequencing. Chemerin-encoding plasmids were transfected into HCAECs using a low pressure-accelerated gene gun (Bioware Technologies, Taipei, Taiwan). The experimental plasmid at 2 μg and control plasmid (pGL4-Renilla luciferase) at 0.02 μg were cotransfected using the gene gun for each, and then the culture medium was replaced with the normal culture medium. After exposure of the cells to hypoxia for 1 h, HCAEC extracts were prepared using the dual luciferase reporter assay system (Promega) and analyzed for dual luciferase activity on a luminometer (Turner Designs).

### Proliferation Assay

The proliferation of HCAECs was quantified using [^3^H]thymidine incorporation. The cells were seeded in ViewPlates (Packard Instrument, Meriden, CT) at a density of 5 × 10^3^/well in a serum-free medium. Thymidine uptake was analyzed by addition of 500 nCi/mL [^3^H]thymidine (Perkin Elmer, Boston, MA) with incubation for 2–6 h with or without hypoxia. The cells were washed twice with PBS. Nonspecific uptake was studied in the presence of 10 μM cytochalasin B and subtracted from the determined values. MicroScint-20 (50 μL) was added, and the plate was read on a TopCount (Packard Instrument).

### Migration Assay

The migration activity of HCAECs was determined using the growth factor-reduced Matrigel invasion system (Becton Dickinson) as previously described.[19] HCAECs (5 × 10^4^ cells) were seeded on top of the ECMatrix gel (Chemicon International, Inc., Temecula, CA) and incubated at 37°C for 4 h during hypoxia with addition of chemerin, TNF-alpha, PD98059, an anti-TNF-alpha antibody, chemerin siRNA, or scrambled siRNA. Three different phase-contrast microscopic high-power fields per well were photographed. The migratory HCAECs were stained with Liu stain (BaSO, New Taipei City, Taiwan) and counted. The observer was blind to the experiment.

### Capillary-Like Network Formation Assay

Capillary-like network formation was tested on cultured HCAECs as previously described [19]. A 24-well culture plate was coated with Matrigel (250 μL; BD Biosciences, MA), which was allowed to solidify (37°C, 1 h). HCAECs were cultured in the plate at hypoxic condition (2.5% O_2_) for 4 h at 37°C. Using a phase contrast microscope, the capillary-like network formation was examined, and its branching points were quantified.

### Statistical Analysis

All results were expressed as mean ± SD. Statistical significance was evaluated using the ANOVA test followed by Tukey–Kramer multiple-comparison test (GraphPad Software Inc., San Diego, CA, USA). Differences with P < 0.05 were considered statistically significant.

## Results

### Hypoxia Increases the Expression of Chemerin in Cultured HCAECs

**Fig 1:**
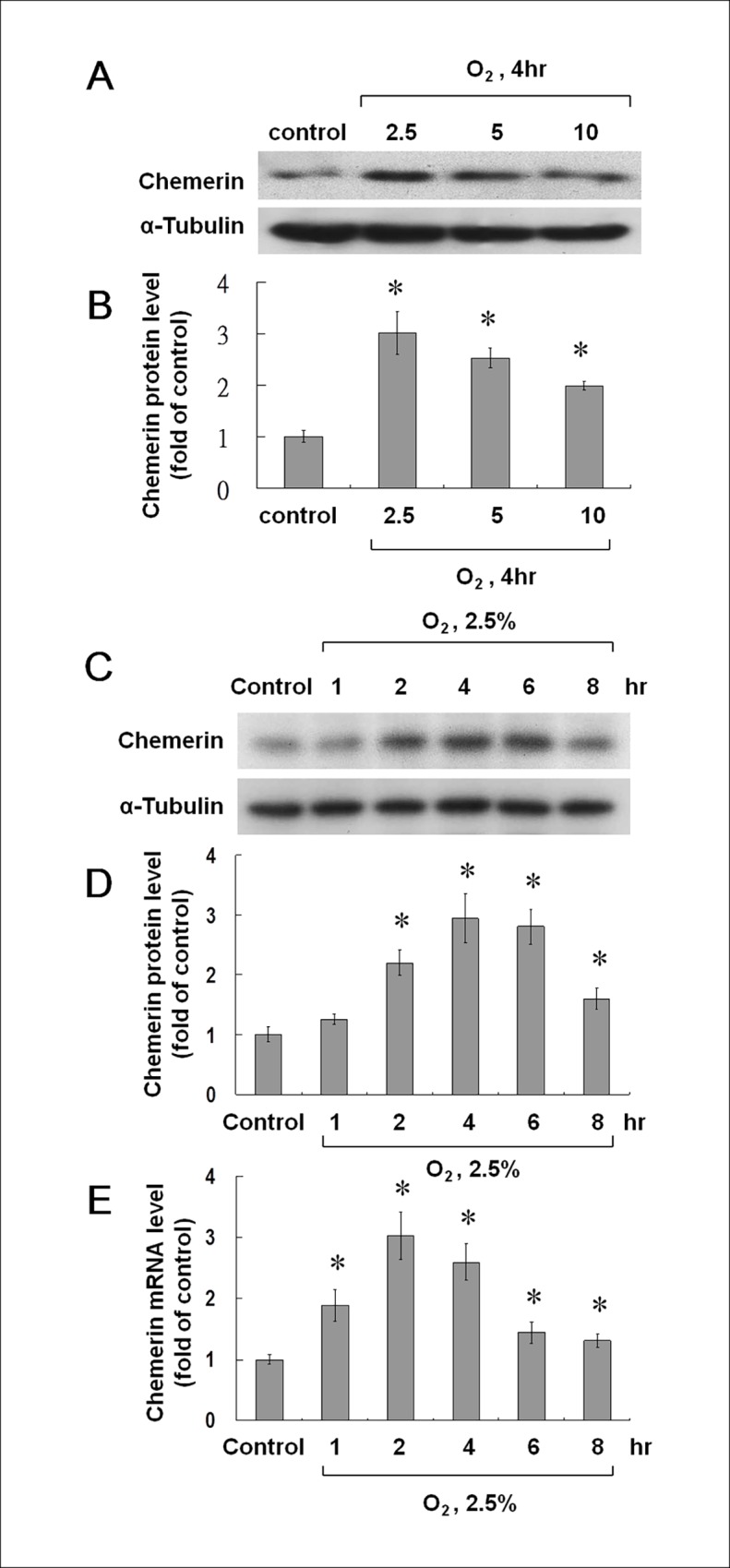
Effects of hypoxia on chemerin protein expression in human coronary artery endothelial cells (HCAECs). (A) A representative western blot of chemerin in HCAECs at different degrees (2.5%, 5% and 10% O_2_) of hypoxia for 4 h. (B) Quantitative analysis of chemerin protein levels (n = 4 per group); (C) Representative western blots for chemerin in cells subjected to 2.5% O_2_ hypoxia for various periods. (D, E) Quantitative analysis of protein and mRNA levels of chemerin. The values from hypoxic HCAECs were normalized to α-tubulin data and then expressed as a ratio of the normalized values to mRNA in control cells (n = 4 per group); *p < 0.01 compared to control.

Different degrees of hypoxia (2.5%, 5%, and 10% O_2_) were used to test the expression of chemerin in cultured HCAECs (Fig 1A and 1B). Chemerin protein expression was notably increased when HCAECs were subjected to 2.5% O_2_ hypoxia for 4 h as compared to other conditions. Thus, these culture conditions were used in subsequent analyses. At 2.5% O_2_ hypoxia, the chemerin level increased gradually and reached a peak after 4 h (Fig 1C and 1D). Chemerin mRNA expression also reached its maximal level after 2 h and decreased gradually (Fig 1E). The pH levels showed no significant differences among the different levels of hypoxia.

### Hypoxia-Induced Chemerin Protein Expression in Cultured HCAECs Is Mediated by TNF-Alpha and the ERK Pathway

**Fig 2:**
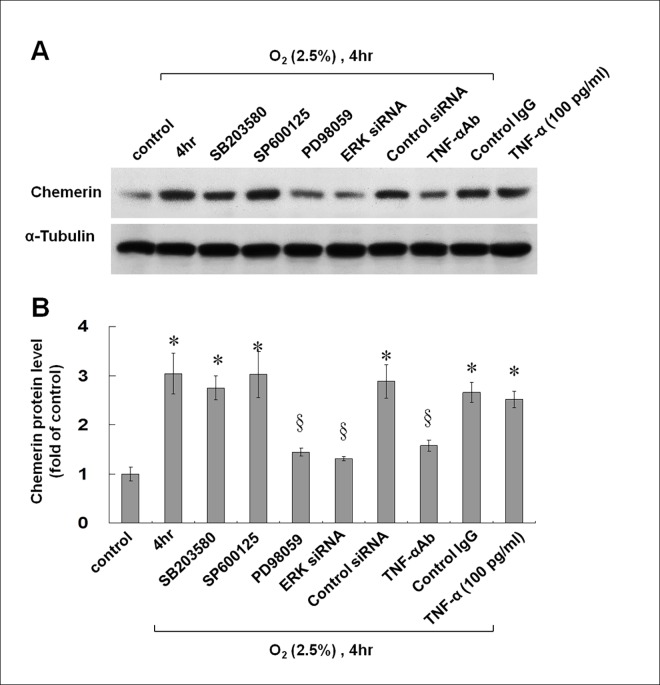
The ERK pathway mediates the hypoxia-induced increase in chemerin expression in HCAECs. (A and B) The hypoxia-induced increase in chemerin expression was blocked by an ERK inhibitor (PD98059), ERK siRNA, or an anti-TNF antibody. Addition of exogenous TNF—alpha during normoxia increased chemerin expression (n = 4 per group); *p < 0.01 compared to control.

To identify the pathways involved in hypoxia-induced chemerin expression, various inhibitors, including inhibitors of p38 MAPK (SB203580), JNK (SP600125), and ERK kinase (PD98059) were used. As shown in Fig 2A and 2B, western blot analysis revealed that the hypoxia-induced chemerin expression was significantly inhibited by the ERK kinase inhibitor (PD98059). This effect was confirmed using an ERK siRNA. The JNK (SP600125) and p38 MAP kinase (SB203580) inhibitors did not attenuate the chemerin protein expression induced by hypoxia (Fig 2A and 2B). These findings indicated that ERK pathways but not p38 MAPK and JNK pathways were responsible for the induction of chemerin expression in hypoxic HCAECs.

TNF-alpha expression was induced by hypoxia at 2.5% O_2_ after 1 h and then decreased gradually (S1 Fig). In addition, pretreatment with an anti-TNF-alpha antibody significantly attenuated the expression of chemerin protein induced by hypoxia, and addition of TNF-alpha increased the expression of chemerin in HCAECs during normoxia (Fig 2A and 2B). These data indicate that TNF-alpha mediates the expression of chemerin by hypoxia.

We also tested the role of HIF-1alpha in HCAECs under hypoxia condition. HIF-1alpha expression was induced by hypoxia at 2.5% after 1 h and then decreased gradually (S2A and S2B Fig). However, addition of HIF-1alpha siRNA did not affect the expression of chemerin protein induced by hypoxia (S2C and S2D Fig).

**Fig 3:**
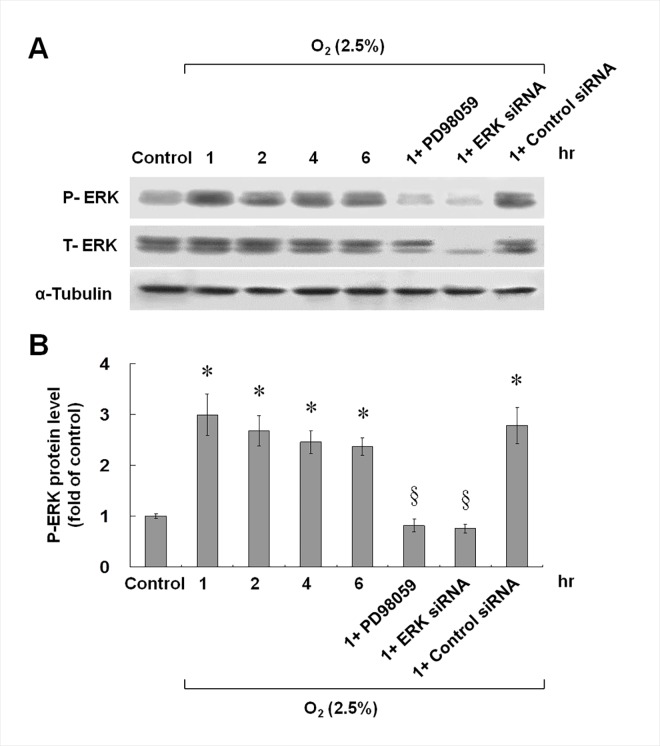
Phosphorylation of ERK was responsible for the hypoxia-induced chemerin expression in HCAECs, which was blocked by PD98059 or ERK siRNA. (A and B) HCAECs were subjected to normoxia or hypoxia for different periods in the presence or absence of the inhibitors. Cell lysates were collected for western blot analysis with an antibody for total ERK and phospho-ERK. T-ERK = total ERK, P-ERK = phosphorylated ERK (n = 4 per group); *p < 0.01 compared to control.

Because the ERK inhibitor reduced the chemerin protein expression most significantly, we focused on the ERK pathway to study the chemerin protein expression in hypoxic HCAECs. Hypoxia at 2.5% O_2_ for 1 to 6 h significantly increased the phosphorylation of ERK (Fig 3A and 3B). The ERK kinase inhibitor (PD98059) and ERK siRNA effectively blocked phosphorylation of the ERK protein induced by hypoxia. The scrambled siRNA did not affect the phosphorylation of ERK induced by hypoxia.

### Hypoxia Increases the Binding of the SP1 Transcription Factor to the Chemerin Promoter and Enhances Transcriptional Activity of SP1 on the Chemerin Promoter in HCAECs

**Fig 4:**
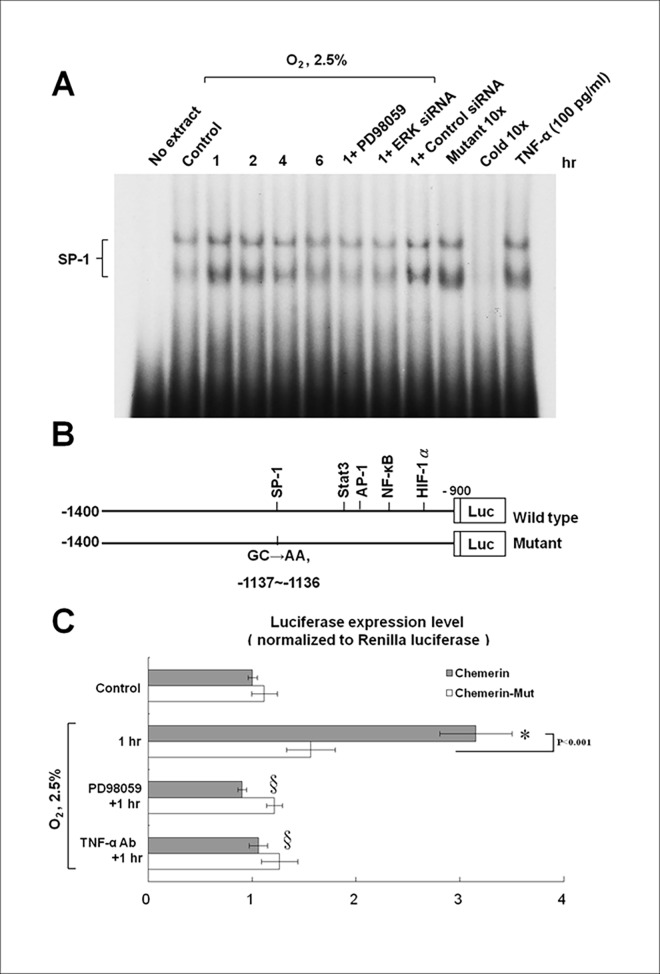
Binding of chemerin promoter with the SP1 transcription factor and transcriptional activity of the SP1-binding site of the chemerin promoter are increased in hypoxic HCAECs. (A) The electrophoretic mobility shift assay (EMSA) showed an increase in binding of chemerin promoter and SP1 in HCAECs during 2.5% O_2_ hypoxia. (B and C) The luciferase reporter assay revealed that hypoxia at 2.5% O_2_ increased the transcriptional activity of SP1 on the chemerin promoter as compared to the chemerin mutant. Transcriptional activity was suppressed by PD98059 and an anti-TNF-alpha antibody (Ab; n = 3 per group); *p < 0.01 compared to control, ^§^p < 0.01 compared to time point 1 h.

Under the conditions of 2.5% O_2_ hypoxia, EMSA showed increased DNA-protein binding activity of SP1 in chemerin promoter (Fig 4A). Addition of PD98059 and ERK siRNA before hypoxia abolished this binding activity. Exogenous addition of TNF-alpha significantly increased the DNA-protein binding activity. This finding suggested that hypoxia in HCAECs may increase the binding of the chemerin promoter with the SP1 transcription factor. Hypoxia-induced ERK phosphorylation may increase chemerin promoter–SP1 binding and chemerin expression.

To study whether the chemerin expression induced by hypoxia is regulated at the transcriptional level, we cloned the promoter region of chemerin and contrasted a luciferase reporter plasmid (pGL3-Luc). The chemerin promoter construct contained SP1-, Stat3-, AP-1, NF-kB, and HIF-1alpha-binding sites. The mutant chemerin promoter has a mutation of SP1-binding site (Fig 4B). The luciferase reporter assay showed that hypoxia at 1 h increased transcriptional activity of SP1 on the chemerin promoter in HCAECs (Fig 4C). Mutation of SP1 binding site in the chemerin promoter inhibited this effect of hypoxia. Addition of PD98059 and the anti-TNF-alpha antibody suppressed the transcriptional activity of the chemerin promoter in hypoxic HCAECs. These results suggest that the SP1 binding site in the chemerin promoter is essential for the transcriptional regulation induced by hypoxia and that hypoxia regulates chemerin promoter via TNF-alpha and ERK pathways.

### PD98059, the Anti-TNF-Alpha Antibody, and Chemerin siRNA Each Inhibit HCAECs Proliferation, Tube Formation, and Migratory Activity Induced by Hypoxia

**Fig 5:**
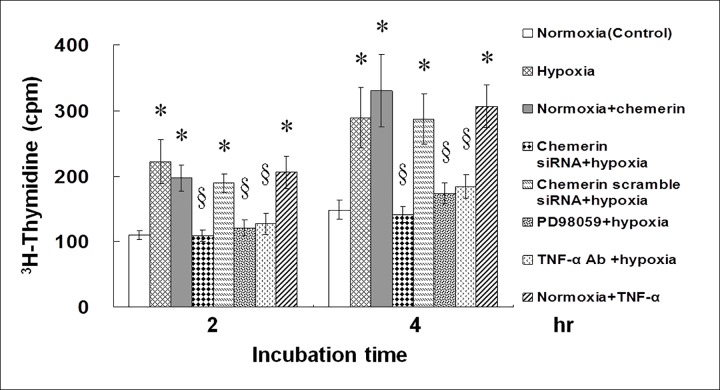
Hypoxia increases proliferation of HCAECs. Incorporation of [^3^H]thymidine into HCAECs increased during 2.5% O_2_ hypoxia and after addition of exogenous chemerin or TNF-alpha during normoxia for 2 to 4 h. Hypoxia-induced incorporation of [^3^H]thymidine into HCAECs was suppressed by chemerin siRNA, PD98059, or by the anti-TNF-alpha antibody (Ab; n = 4 per group); *p < 0.01 compared to control, ^§^p < 0.01 compared to hypoxia. Cpm: counts per minute.

To test the effects of hypoxia on the function of HCAECs, we examined HCAECs proliferation, tube formation, and migratory activity. In addition, chemerin siRNA (S3 Fig) was designed to test the role of chemerin in HCAECs. Proliferation of HCAECs was evaluated by measuring ^3^H-thymidine incorporation into the cells. Results revealed an increase in HCAEC proliferation after 2 and 4 h of hypoxia (Fig 5). Pretreatment with chemerin siRNA, PD98059, and the anti-TNF-alpha antibody attenuated the proliferation induced by hypoxia. Exogenous addition of chemerin and TNF-alpha during normoxia also increased proliferation of HCAECs.

**Fig 6:**
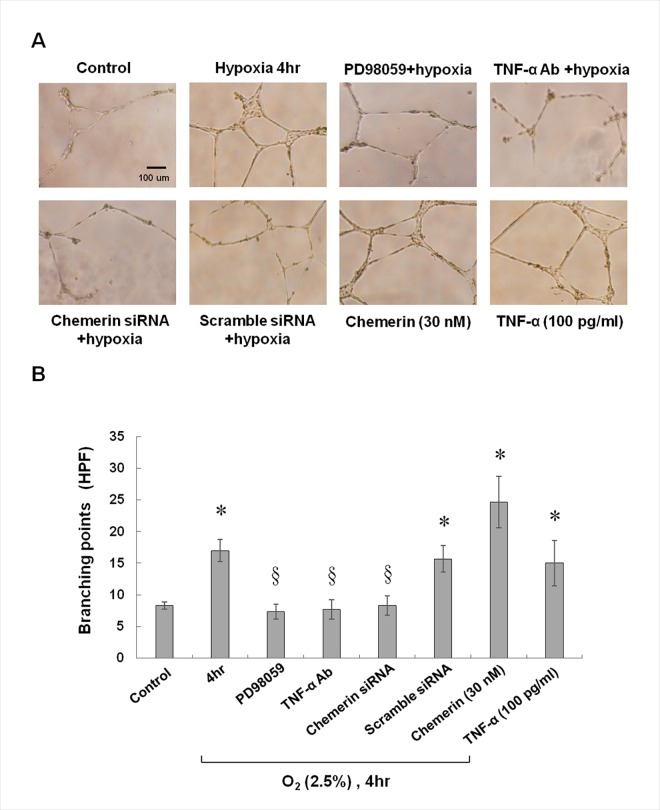
Effects of hypoxia on tube formation by HCAECs. (A) A representative image of a tube formed by HCAECs. (B) Quantitative branching points analysis (n = 4 per group); *p < 0.01 compared to control, ^§^p < 0.01 compared to hypoxia.

Hypoxia at 2.5% O_2_ significantly increased HCAEC migration (S4 Fig) and capillary-tube formation (by counting the branching point) (Fig 6) at 4 h as compared with the control group. Addition of PD98059, the anti-TNF-alpha antibody, or chemerin siRNA attenuated the enhancement of HCAEC migration and capillary tube formation induced by 4 h of hypoxia. The control siRNA did not inhibit the enhancement of migration and capillary tube formation induced by hypoxia. Addition of chemerin and TNF-alpha increased the HCAEC migration and capillary-tube formation during normoxia.

## Discussion

We currently report the following major findings: First, hypoxia induces chemerin expression in cultured HCAECs in a time- and dose-dependent manner. Second, TNF-alpha acts as an autocrine factor that mediates hypoxia-induced chemerin protein expression in HCAECs. Third, ERK and transcription factor SP1 are involved in the signaling pathways driving the chemerin induction by hypoxia. Fourth, hypoxia increases migratory activity of (and tube formation by) HCAECs, and these effects are at least partially dependent on chemerin.

Chemerin plays an important role in cardiovascular diseases. One study showed that chemerin mRNA and protein levels are elevated in the epicardial adipose tissue from patients with ischemic heart disease, where these parameters correlate with the severity of coronary atherosclerotic change [5]. In addition, serum chemerin levels are elevated in patients with coronary artery disease and acute coronary syndrome [23] and correlate with the severity and extent of coronary artery stenosis [5, 24]. In the present study, our aim was to determine whether chemerin could affect the proliferation and migration of coronary endothelial cells.

Our study showed that TNF-alpha, a pro-inflammatory cytokine, exerted a potent effect on the chemerin expression during normoxia. In addition, hypoxia induced HCAECs to secrete TNF-alpha, and administration of anti-TNF-alpha antibody attenuated the increase in chemerin expression by hypoxia. This finding suggest TNF-alpha acts as an autocrine factor in mediating hypoxia-induced chemerin expression in HCAECs, which is consistent with those from another study, which showed that inhibition of TNF-alpha lowers chemerin levels in patients with active rheumatoid arthritis [25].

We tested the involvement of three signaling pathways in chemerin expression. Our study showed that JNK, p38 MAPK, and HIF-1alpha do not have any effect on chemerin expression, but ERK has a potent effect on this expression. SP1, a well characterized downstream target of ERK, is the main transcriptional factor activated by TNF-alpha. In the present study, we demonstrated that hypoxia increased SP1 binding to a relevant promoter (chemerin gene). The anti-TNF-alpha antibody and the ERK inhibitor PD98059 inhibited this SP1 binding induced by hypoxia, indicating that TNF alpha and the ERK pathway mediate the increased transcriptional regulation driven by SP1 in the hypoxic model. In addition, the anti-TNF alpha antibody and the ERK inhibitor (PD98059) attenuated the enhancement of chemerin promoter activity by hypoxia. In this study, we also demonstrated that the SP1-binding site in the chemerin promoter is essential for the transcriptional regulation by hypoxia, because a mutant SP1-binding site in the chemerin promoter abrogated the transcriptional activity induced by hypoxia.

In the present study, we found that hypoxia for 4 h increased HCAEC proliferation, tube formation, branching and migration, which are the early processes of angiogenesis. This indicates that coronary artery angiogenesis may increase in ischemic heart disease. Exogenous addition of chemerin also increased migration and proliferation of HCAECs without hypoxia. Chemerin siRNA, PD98059, and TNF-alpha, each attenuated the migration and proliferation of HCAECs induced by hypoxia. These findings suggest that in order to counteract the effects of hypoxia, chemerin-driven angiogenesis is increased in HCAECs, and this effect is mediated by TNF-alpha and ERK.

## Conclusion

Our study shows for the first time that hypoxia enhances chemerin expression in cultured HCAECs. The hypoxia-induced chemerin expression is mediated by TNF-alpha and at least in part by the ERK pathway. Chemerin increases capillary-tube formation as well as migratory activity of HCAECs after hypoxic treatment, indicating that chemerin counteracts the ischemic effects of hypoxia. Thus, chemerin may be a novel therapeutic target in ischemic heart disease for interventions intended to increase angiogenesis (S5 Fig).

## Supporting Information

S1 Fig Representative western blots of TNF-alpha in HCAECs subjected to 2.5% O_2_ hypoxia for various periods. (TIF)

S2 Fig Effects of hypoxia on hypoxia induced factor-1alpha (HIF-1alpha) protein expression in HCAECs. (A and B) Representative western blots and quantitative analysis for HIF-1alpha protein level in HCAECs subjected to 2.5% O_2_ hypoxia for various periods. (C and D) The effect of HIF-1alpha siRNA on chemerin protein expression in HCAECs under hypoxia. (n = 4 per group); The values from hypoxic HCAECs were normalized to the data on α-tubulin. *p < 0.01 compared to control.
(TIF)

S3 Fig Effects of chemerin siRNA on chemerin protein expression. (A) Representative western blots of chemerin in cells subjected to 2.5% O_2_ hypoxia; effects of chemerin siRNA and scrambled siRNA. (B) Quantitative analysis of chemerin. The values from hypoxic HCAECs were normalized to the data on α-tubulin (n = 4 per group); *p < 0.01 compared to control, ^§^p < 0.01 compared to hypoxia.
(TIF)

S4 Fig Hypoxia increases migratory activity of HCAECs. (A and B) Hypoxia at 2.5% O_2_ for 4 h increased migratory activity of HCAECs, which was inhibited by PD98059, an anti-TNF-alpha antibody (Ab), or chemerin siRNA. Furthermore, addition of exogenous chemerin or TNF-alpha during normoxia also increased migratory activity of HCAECs (n = 4 per group); *p < 0.01 compared to control, ^§^p < 0.01 compared to hypoxia.
(TIF)

S5 Fig A schematic representation of the TNF-alpha and ERK pathways involved in the hypoxia-induced induction of chemerin expression leading to HCAEC-mediated angiogenesis via proliferation and migration of these cells after transcriptional changes. Ab: antibody.
(TIF)

## Abbreviations

ATP: Adenosine triphosphate
CMKLR1: Chemokine like receptor 1
EMSA: Electrophoretic mobility shift assay
HCAECs: Human coronary artery endothelial cells
HIF-1alpha: Hypoxia induce factor-1alpha
IL-6: Interleukin 6
JNK: Jun N-terminal kinase
MAPK: Mitogen-activated protein kinase
siRNA: Short interfering RNA
TNF-alpha: Tumor necrosis factor-alpha
