# Supplementary figures and images for: Tumor Necrosis Factor-Alpha and the ERK Pathway Drive Chemerin Expression in Response to Hypoxia in Cultured Human Coronary Artery Endothelial Cells
Source: PLoS One. 2016 Oct 28;11(10):e0165613. doi: 10.1371/journal.pone.0165613 (PMC5085022; doi:10.1371/journal.pone.0165613)

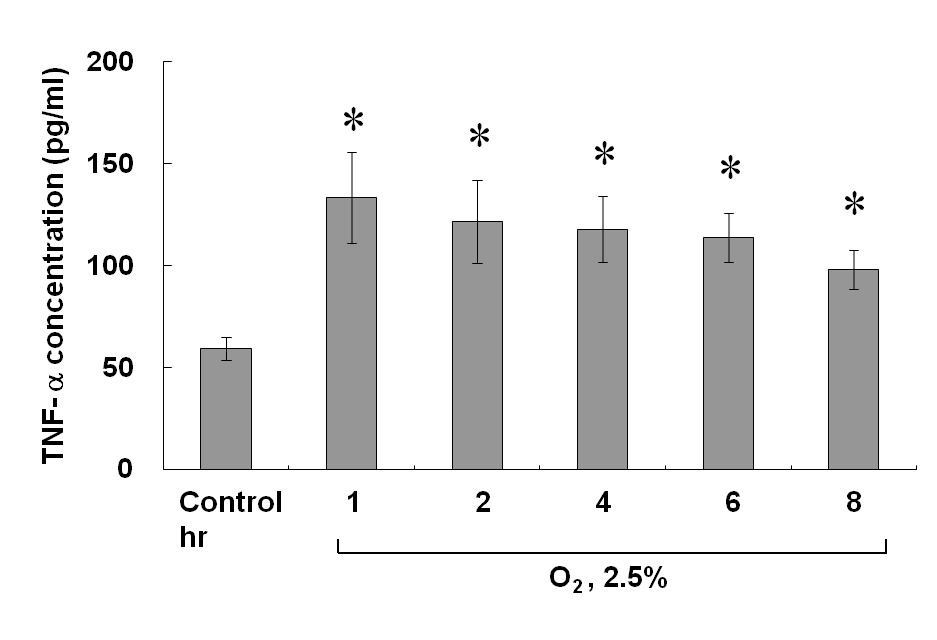

Supplement: S1 Fig — (TIF) [file pone.0165613.s001.tif]

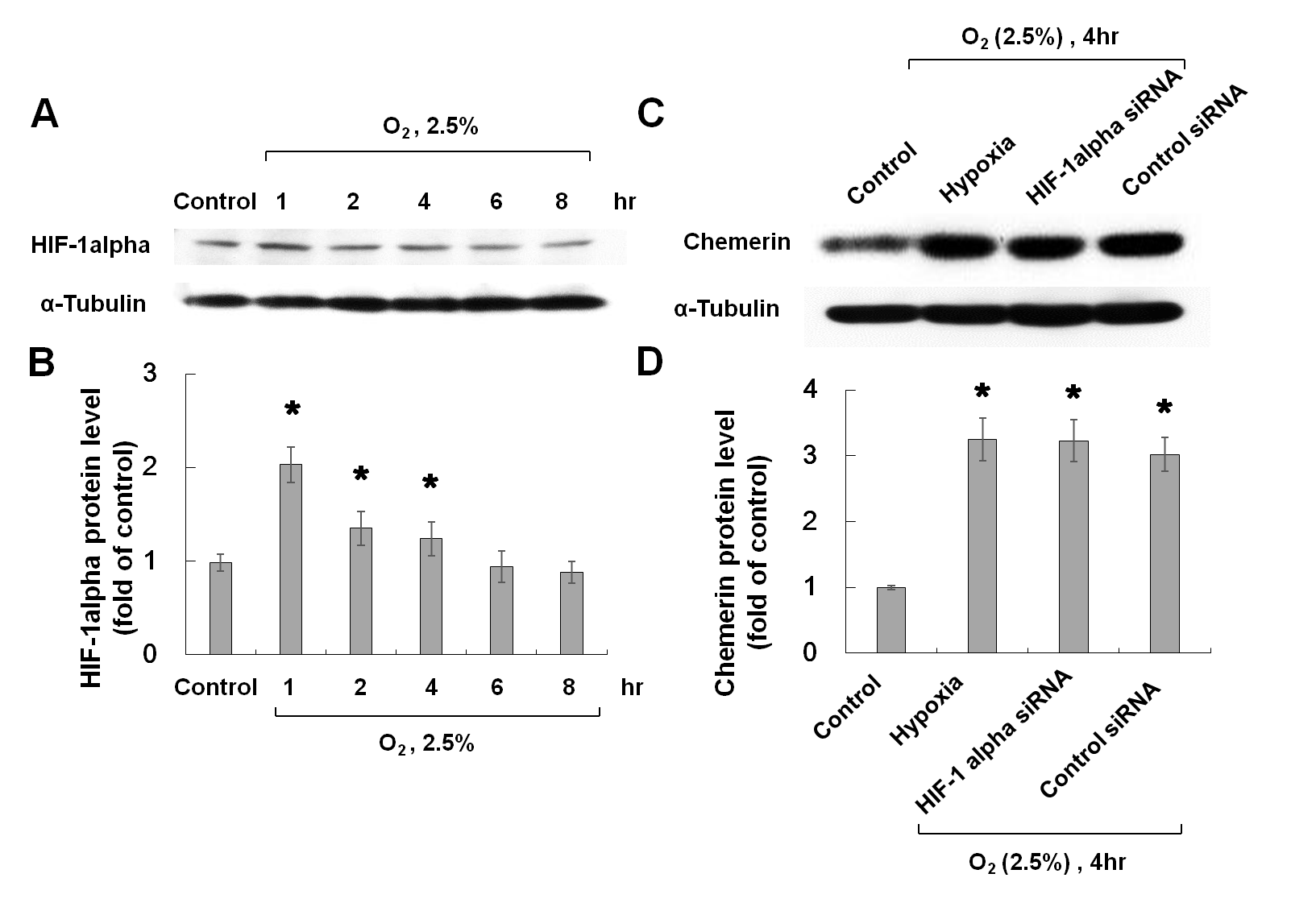

Supplement: S2 Fig — (A and B) Representative western blots and quantitative analysis for HIF-1alpha protein level in HCAECs subjected to 2.5% O2 hypoxia for various periods. (C and D) The effect of HIF-1alpha siRNA on chemerin protein expression in HCAECs under hypoxia. (n = 4 per group); The values from hypoxic HCAECs were normalized to the data on α-tubulin. *p < 0.01 compared to control. (TIF) [file pone.0165613.s002.tif]

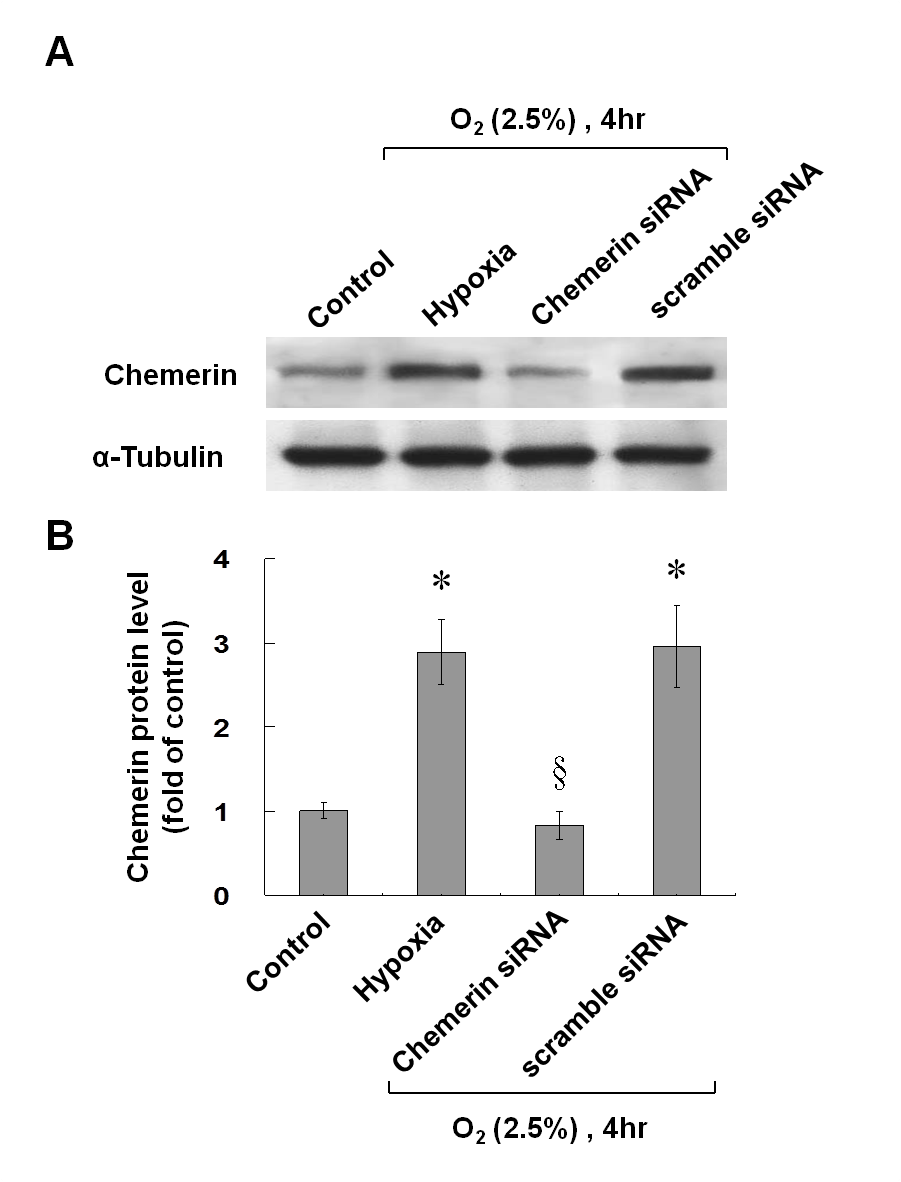

Supplement: S3 Fig — (A) Representative western blots of chemerin in cells subjected to 2.5% O2 hypoxia; effects of chemerin siRNA and scrambled siRNA. (B) Quantitative analysis of chemerin. The values from hypoxic HCAECs were normalized to the data on α-tubulin (n = 4 per group); *p < 0.01 compared to control, §p < 0.01 compared to hypoxia. (TIF) [file pone.0165613.s003.tif]

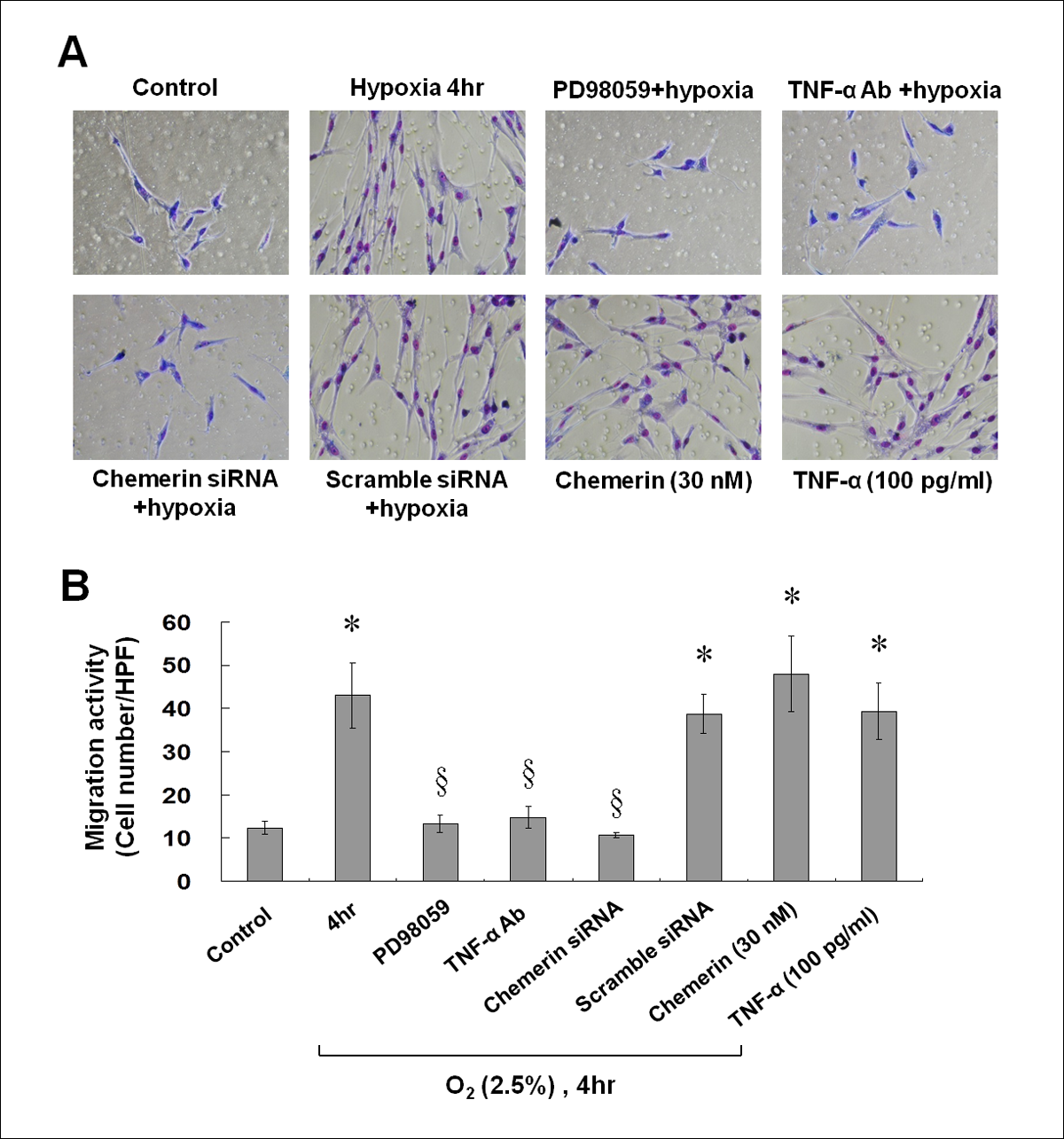

Supplement: S4 Fig — (A and B) Hypoxia at 2.5% O2 for 4 h increased migratory activity of HCAECs, which was inhibited by PD98059, an anti-TNF-alpha antibody (Ab), or chemerin siRNA. Furthermore, addition of exogenous chemerin or TNF-alpha during normoxia also increased migratory activity of HCAECs (n = 4 per group); *p < 0.01 compared to control, §p < 0.01 compared to hypoxia. (TIF) [file pone.0165613.s004.tif]

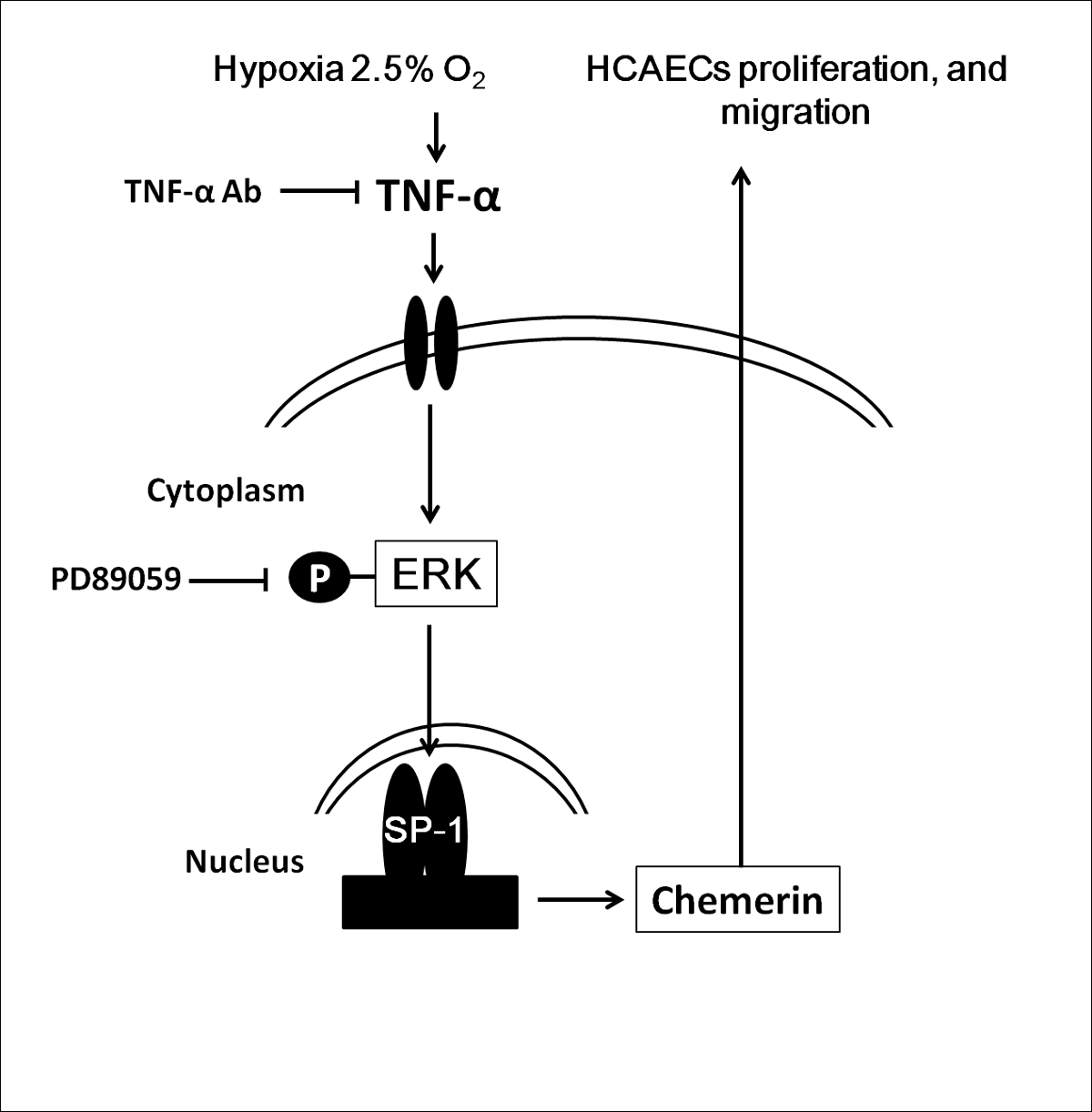

Supplement: S5 Fig — Ab: antibody. (TIF) [file pone.0165613.s005.tif]
